# Implications of Artificial Intelligence in Addressing Antimicrobial Resistance: Innovations, Global Challenges, and Healthcare’s Future

**DOI:** 10.3390/antibiotics13060502

**Published:** 2024-05-29

**Authors:** Francesco Branda, Fabio Scarpa

**Affiliations:** 1 Unit of Medical Statistics and Molecular Epidemiology, Università Campus Bio-Medico di Roma, 00128 Rome, Italy; 2 Department of Biomedical Sciences, University of Sassari, 07100 Sassari, Italy

**Keywords:** artificial intelligence, antibiotic resistance, machine learning, genomic analysis, data quality, interdisciplinary collaboration, decision support systems, antibiotic discovery, model interpretability

## Abstract

Antibiotic resistance poses a significant threat to global public health due to complex interactions between bacterial genetic factors and external influences such as antibiotic misuse. Artificial intelligence (AI) offers innovative strategies to address this crisis. For example, AI can analyze genomic data to detect resistance markers early on, enabling early interventions. In addition, AI-powered decision support systems can optimize antibiotic use by recommending the most effective treatments based on patient data and local resistance patterns. AI can accelerate drug discovery by predicting the efficacy of new compounds and identifying potential antibacterial agents. Although progress has been made, challenges persist, including data quality, model interpretability, and real-world implementation. A multidisciplinary approach that integrates AI with other emerging technologies, such as synthetic biology and nanomedicine, could pave the way for effective prevention and mitigation of antimicrobial resistance, preserving the efficacy of antibiotics for future generations.

## 1. Introduction

**Figure 1:**
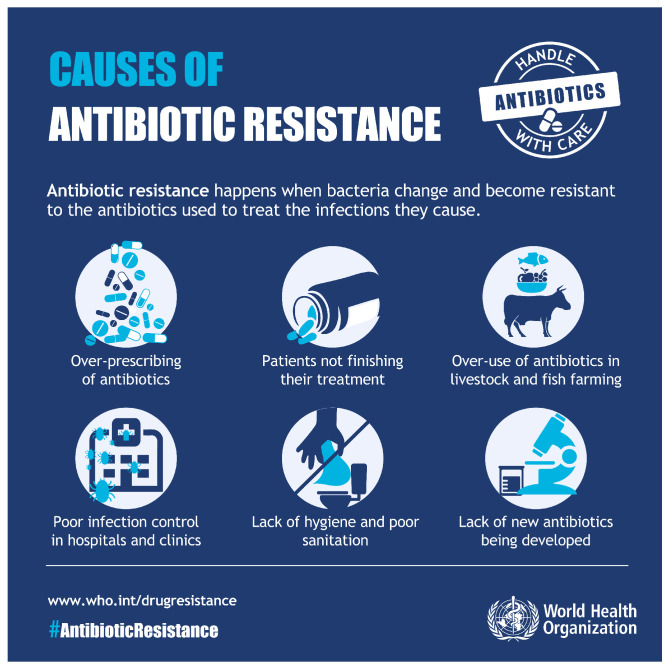
Key causes of antibiotic resistance: over-prescribing, patients not finishing treatment, overuse in livestock and fish farming, poor infection control in hospitals, lack of hygiene and sanitation, and lack of new antibiotics development. Source: WHO, available at https://www.who.int/europe/multi-media/item/causes-of-antibiotic-resistance (accessed on 25 May 2024).

Antibiotic resistance stems from a complex interplay between intrinsic bacterial biology and external influences related to human activities (Figure 1). Understanding these causes is crucial for developing effective strategies against it. Resistance primarily arises from bacterial adaptation and evolution in response to excessive and improper antibiotic use [1]. This is driven by factors such as inappropriate administration in human and veterinary medicine, noncompliance with medical prescriptions, and widespread use in intensive farming, as well as inadequate hygiene in healthcare settings. The intrinsic ability of bacteria to mutate rapidly and transfer resistance genes among themselves through mechanisms such as transformation, transduction, and conjugation further contributes to its spread [2]. Antibiotic resistance poses a significant threat to global public health and healthcare systems, compromising the effectiveness of antibiotic treatments and increasing morbidity and mortality associated with bacterial infections. Infections caused by resistant bacteria require longer and more expensive treatments, escalating healthcare costs and impeding patient recovery. Moreover, resistance can prolong hospital stays, leading to overcrowding and increasing the risk of nosocomial infection transmission. This global crisis jeopardizes the treatment of various diseases, imperiling millions of lives and necessitating urgent action [3]. Indeed, formerly manageable illnesses such as pneumonia, urinary tract infections, and skin infections now require more aggressive and costly treatments due to antibiotic resistance [4]. The effectiveness of antibiotics in critical medical procedures such as surgeries and organ transplants is compromised as well.

To effectively address the growing threat of antibiotic resistance, emerging technologies such as artificial intelligence (AI) offer unprecedented opportunities to improve understanding and response to this phenomenon [5]. AI is revolutionizing the process of drug discovery and development, enabling the efficient exploration of vast virtual chemical spaces and accelerating the identification of new potentially therapeutic molecules [6,7]. Machine learning and deep learning algorithms can analyze huge amounts of data, identifying complex patterns and filtering candidate molecules based on desirable properties [8]. One of the most promising approaches is the computational design of new antibiotics [9], in which machine learning models analyze the molecular structures of existing antibiotics and their protein targets to identify potential modifications that can overcome bacterial resistance mechanisms [10]. In addition, AI can be used to identify new therapeutic targets by exploiting the growing genomic and transcriptional data on resistant bacteria. Despite the challenges related to the need for high-quality data and experimental validation, the integration of AI into new drug research offers significant potential for combating antibiotic resistance [11]. Despite the many opportunities offered by AI, it is important to recognize that its integration into new drug research still presents significant challenges. In addition to the need for high-quality data and experimental validation of computational results, the transparency and interpretability of machine learning algorithms remain a priority to ensure the safety and efficacy of newly developed drugs [12].

Veterinary medicine faces similar challenges, impacting livestock, companion animals, and wildlife [13]. The connection between human and animal health is undeniable, with the reduced effectiveness of antibiotics against resistant bacteria presenting obstacles in the management and containment of infectious diseases among livestock, companion animals, and wildlife [14]. Epidemiological trends indicate a steady rise in infections caused by resistant bacteria over recent decades, making antibiotic resistance a major global health threat. This paper examines the fundamental aspects of antimicrobial resistance (AMR) and the role of AI in addressing AMR, highlighting the innovative applications, current challenges, and future prospects of this emerging technology in pharmaceutical and healthcare research. Specifically, Section 2 describes how genetic mutations in bacteria, which alter antibiotic targets or uptake/expulsion mechanisms, are a major cause of the emergence of resistance. Section 3 illustrates the crucial role of horizontal gene transfer in the rapid spread of resistance genes among bacterial populations through mechanisms such as conjugation, transformation, and transduction. Section 4 delves into the importance of plasmids and other mobile genetic elements in carrying and disseminating antibiotic resistance genes among bacteria. Section 5 describes how AI can analyze large volumes of data, such as microscopic images of bacterial cultures and genomic data, to rapidly identify markers of antibiotic resistance. Section 6 illustrates the potential of AI-based decision support systems to guide physicians’ antibiotic prescribing by analyzing clinical data and microbial profiles of patients. Section 7 examines the application of AI in the process of discovering new antibiotics, from virtual screening of chemical compounds to de novo design of molecules and optimization of candidates through structure–activity relationship analysis. Finally, Section 8 concludes the paper, emphasizing that AMR requires a multidisciplinary approach. It is critical to integrate the understanding of molecular mechanisms with the development of innovative strategies in order to preserve the efficacy of antibiotics. Without effective interventions, future projections suggest a dramatic increase in difficult-to-treat infections and mortality rates. Therefore, adopting preventive and control measures to contain the spread of antibiotic resistance and preserve the effectiveness of antibiotics for future generations is imperative.

## 2. Genetic Mutations Associated with Antibiotic Resistance

Genetic mutations represent one of the primary causes of the emergence of antibiotic resistance, contributing to genetic diversification and bacterial survival in the presence of antimicrobial agents [15]. These mutations can affect a wide range of genes, including those involved in antibiotic action mechanisms and those related to their penetration into bacterial cells, efflux, or inactivation [16]. One of the most common ways for bacteria to develop resistance to antimicrobials is represented by mutations in the antibiotic target genes [17]. For instance, in Gram-positive bacteria, mutations in genes encoding penicillin-binding proteins (PBPs) can alter the structure of target proteins, reducing the antibiotic’s affinity for its binding site and rendering the bacterium resistant to its action. Similarly, in Gram-negative bacteria, mutations in genes encoding porins, the channels for antibiotic entry into the bacterial cell, can reduce the effectiveness of the antibiotic in crossing the outer membrane and reaching its site of action [18]. Mutations in genes involved in antibiotic transport and efflux mechanisms can influence the bacterium’s ability to absorb the antibiotic from the surrounding environment or to expel it once absorbed [19]. For example, mutations in genes encoding efflux pumps can increase the activity of these proteins, allowing the bacterium to expel the antibiotic before it can exert its antibacterial effect [20]. Additionally, mutations in genes encoding nutrient transporters can indirectly affect antibiotic absorption by altering the composition and permeability of the bacterial cell membrane [21]. In antibiotic inactivation systems, mutations can allow some bacteria to develop resistance to antibiotics through mutations in genes encoding enzymes capable of inactivating the antibiotic before it can exert its antibacterial effect. For example, Gram-negative bacteria such as *Escherichia coli* can develop resistance to penicillin through mutations in genes encoding beta-lactamases, enzymes that degrade the beta-lactam ring of the antibiotic, rendering it ineffective in binding to PBPs and blocking cell wall synthesis [22]. In general, genetic mutations represent an adaptive response of bacteria to antibiotics, allowing them to survive and proliferate in the presence of antimicrobial selective pressures. The diversity and plasticity of the bacterial genome provide multiple evolutionary pathways for antibiotic resistance development, emphasizing the importance of a thorough understanding of the underlying genetic mechanisms to effectively address this growing issue.

## 3. Horizontal Transfer of Resistance Genes among Bacteria

Horizontal gene transfer (HGT) stands as a pivotal mechanism in the dissemination of antibiotic resistance among bacterial populations [23]. Unlike vertical transmission, which involves the transfer of genetic material from parent to offspring, HGT allows for the exchange of genetic information between bacteria of different species or strains. This phenomenon significantly contributes to the rapid spread and acquisition of antibiotic resistance traits, posing a formidable challenge to antimicrobial therapy and public health [24]. Conjugation, one such mechanism, involves the direct transfer of genetic material through physical contact between donor and recipient cells [25]. Conjugation is facilitated by conjugative plasmids, which are extrachromosomal DNA molecules capable of self-replication and transfer between bacterial cells [26]. These plasmids often carry genes encoding antibiotic resistance determinants, such as beta-lactamases, efflux pumps, or target-modifying enzymes. Accordingly, conjugative plasmids can transfer not only resistance genes but also virulence factors and other adaptive traits, further complicating the management of bacterial infections. Another mechanism of HGT is transformation, wherein bacteria take up free DNA molecules released into the environment by lysed cells or during cellular degradation [27]. This process allows bacteria to acquire new genetic material, including antibiotic resistance genes, from surrounding microbial communities. Transformation is particularly relevant in natural environments, where bacterial populations are exposed to diverse genetic pools and selective pressures, fostering the exchange of genetic information and the emergence of novel resistance phenotypes. Furthermore, bacteriophages, which are viruses that infect bacteria, play a crucial role in HGT through a process known as transduction. During transduction, bacteriophages inadvertently package bacterial DNA fragments, including antibiotic resistance genes, into their viral capsids during the lytic cycle [28]. Upon infecting new bacterial hosts, these phages inject their genetic cargo, thereby transferring resistance traits between bacteria [29]. The widespread occurrence of HGT underscores its significance in shaping bacterial evolution and adaptation to environmental challenges, including antibiotic exposure. By promoting genetic diversity and facilitating the acquisition of adaptive traits, HGT enables bacteria to rapidly respond to selective pressures and overcome antimicrobial interventions [30]. Unfortunately, the rampant dissemination of antibiotic resistance genes through HGT poses a serious threat to public health, complicating treatment strategies and limiting the effectiveness of available antibiotics. In addition, Outer Membrane Vesicles (OMVs) are emerging as a pathway for the transfer of genetic material and proteins that plays a significant role in the spread of antibiotic resistance. In particular, Neisseria gonorrhoeae has demonstrated the ability to exploit these structures to transfer its resistance genes, thereby contributing to the rapid dissemination of antibiotic-resistant strains. OMVs, which are small membrane sacs released by bacterial cells, can contain a variety of biologically active molecules, including antibiotic resistance genes. This mechanism of gene transfer provides an additional avenue through which pathogens can acquire and spread antibiotic resistance [31].

## 4. Role of Plasmids and Mobile Genetic Elements in Resistance Spread

The role of plasmids and mobile genetic elements in spreading antibiotic resistance is crucial in the epidemiology and evolution of bacterial resistance to antibiotics. Plasmids are small extrachromosomal DNA fragments that are primary vehicles for transferring antibiotic resistance genes between bacteria [32]. These genetic elements can replicate autonomously within bacterial cells, and are transmitted through processes such as conjugation, transformation, and transduction [33]. Additionally, mobile genetic elements such as transposons, integrons, and phages significantly contribute to resistance dissemination. Transposons move within the bacterial genome, carrying resistance genes, while integrons capture and integrate new resistance genes, allowing rapid acquisition of new determinants. Phages are viruses that infect bacteria; they can transfer resistance genes during infection cycles, spreading resistance across strains and species. The interaction between plasmids, transposons, integrons, and phages creates a dynamic genetic environment fostering the emergence and spread of antibiotic resistance. This genetic flexibility enables bacteria to quickly adapt to antibiotic pressures, posing a serious threat to public health. Understanding the role and impact of these elements is essential for developing strategies to control and mitigate antibiotic resistance while ensuring the efficacy of antibiotics for future generations.

## 5. Artificial Intelligence-Based Diagnostic Tools for Early Detection of Antibiotic Resistance

**Figure 2:**
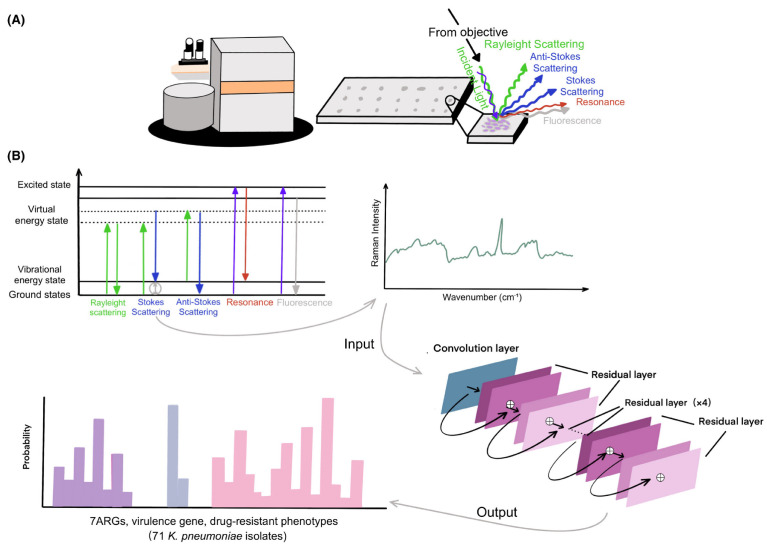
Schematic overview of confocal Raman microscopy techniques, from sample preparation to spectral analysis and construction of the ResNet taxonomic model [37]. (**A**) illustrates the experimental setup with a confocal Raman microscope analyzing a bacterial sample, showing the various types of scattering and fluorescence phenomena that can be observed, while (**B**) presents a diagram of the vibrational energy levels and electronic states involved in Raman scattering phenomena, with a typical Raman spectrum resulting. Using a one-dimensional residual network with 25 total convolutional layers, Raman spectra are analyzed to predict the existence of ARGs and virulence genes or drug-resistant phenotypes.

Antibiotic resistance represents one of the greatest challenges to global public health, as it reduces the effectiveness of antibiotic treatments and increases the risk of difficult-to-treat infections. In this context, artificial intelligence (AI)-based diagnostic tools can analyze large volumes of data, often with greater accuracy than humans, enabling faster and more targeted diagnoses. One of the main applications of AI in the diagnosis of antibiotic resistance is the analysis of microscopic images. Machine learning algorithms can be trained to recognize specific features in images [34] of bacterial cultures that are indicative of antibiotic resistance. For example, a study conducted by Hayashi et al. [35] focused on using Convolutional Neural Networks (CNNs) to identify drug-resistant bacterial cells in transmission electron microscope (TEM) images without the need for antibiotic exposure. Their study revealed that drug-resistant strains maintain morphological changes even in the absence of drugs, suggesting a link between genetic changes during the acquisition of drug resistance and morphological changes. CNNs and Support Vector Machines (SVMs) have been widely explored for their ability to identify complex patterns in microbiological and genetic data [36,37], as shown in Figure 2, which schematically illustrates the use of confocal Raman microscopy techniques for spectral analysis and classification of antibiotic-resistant bacteria using a Residual Neural Network model (ResNet).

**Figure 3:**
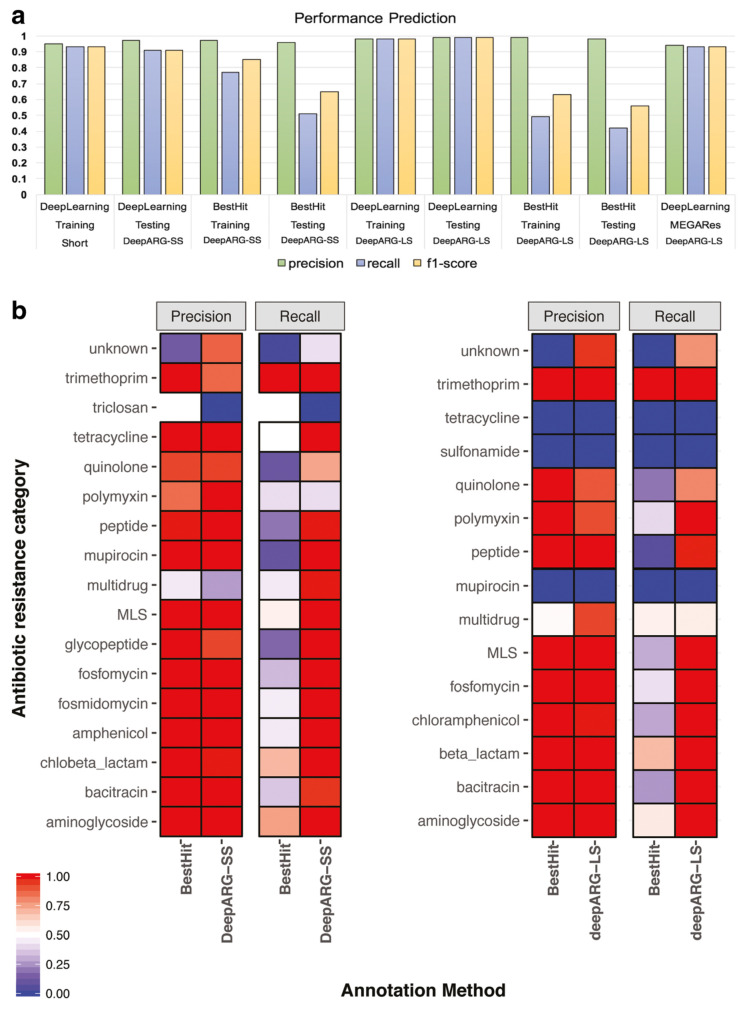
(**a**) Performance comparison of DeepARG models with the best-hit approach using precision, recall, and F1-score as metrics for the training and test datasets. MEGARes bars represent the performance of DeepARG-LS using genes from the MEGARes database. (**b**) Precision and recall of DeepARG models compared with the best hit approach for each individual category in the test dataset [41].

A significant example is the use of deep learning algorithms to rapidly analyze antibiotic susceptibility testing (AST) [38], which can reduce the time needed to determine resistance from days to a few hours. Recurrent Neural Networks (RNNs) contribute significantly to antibiotic resistance diagnostics. For example, RNNs excel at processing sequential data, making them valuable in time series analysis of antibiotic treatments and bacterial growth patterns [39]. Genomic data analysis represents another frontier where AI is demonstrating significant impact. Machine learning algorithms, ranging from Decision Tree (DT) to Gradient Boosting Machines (GBMs), play a key role in the discovery of genetic markers associated with antibiotic resistance [40]. For example, Arango-Argoty et al. [41] developed DeepARG, a novel deep learning approach designed to predict ARGs from metagenomic data, which consists of two models to respond to different annotation strategies, i.e., DeepARG-SS for short read sequences and DeepARG-LS for full gene length sequences. As demonstrated in Figure 3, DeepARG models tend to perform better than the best-hit approach for most categories, which often results in a high false-negative rate, demonstrating the effectiveness of deep learning in identifying resistance genes from genomic data. In addition to DeepARG, other machine learning approaches, such as Random Forest (RF), have been employed to analyze genomic data for antibiotic resistance genes [42]. These models leverage large-scale genomic databases to identify genetic traits associated with resistance, providing valuable insights into the mechanisms that drive antibiotic resistance. AI techniques are increasingly being applied to predict bacterial phenotypes from genomic data, including antibiotic susceptibility profiles [43]. By integrating genomic information into machine learning models, researchers can predict the likelihood of bacterial strains exhibiting resistance to specific antibiotics, facilitating more informed therapeutic decisions in clinical settings. Despite these advances, challenges persist in applying AI to the diagnosis of antibiotic resistance. A significant obstacle is the availability of high-quality well-annotated data, which is essential for effectively training machine learning algorithms [44].

Although there is an abundance of data in various forms, including genomic sequences, medical records, and bacterial culture images, much of these data lack adequate annotation and standardization. This deficiency prevents the development of accurate and reliable artificial intelligence models for predicting antibiotic resistance. AI systems must be able to handle the incomplete and/or noisy data commonly encountered in the real clinical world [45]. Variability in data quality, sample collection procedures, and laboratory techniques can introduce bias and confounding factors that can affect the performance of AI models. Moreover, ensuring the robustness and interpretability of AI systems remains a critical concern [46]. AI algorithms are inherently complex, which makes it difficult to understand how they arrive at their predictions, especially in medical settings, where interpretability is critical to gaining the trust of physicians and patients. Addressing these challenges requires interdisciplinary collaboration among clinicians, data scientists, and experts in the field. Efforts to curate and annotate high-quality datasets are essential for training robust AI models that can accurately predict antibiotic resistance. In addition, research on explainable AI techniques is critical to improving the interpretability of AI systems, enabling clinicians to understand and trust the decisions made by these models [47]. Advances in data preprocessing and algorithm development are needed to ensure that AI systems can effectively handle noisy and incomplete data in order to ultimately improve their performance in real-world clinical settings.

## 6. Optimizing Antibiotic Use through Artificial Intelligence-Guided Decision Support Systems

**Figure 4:**
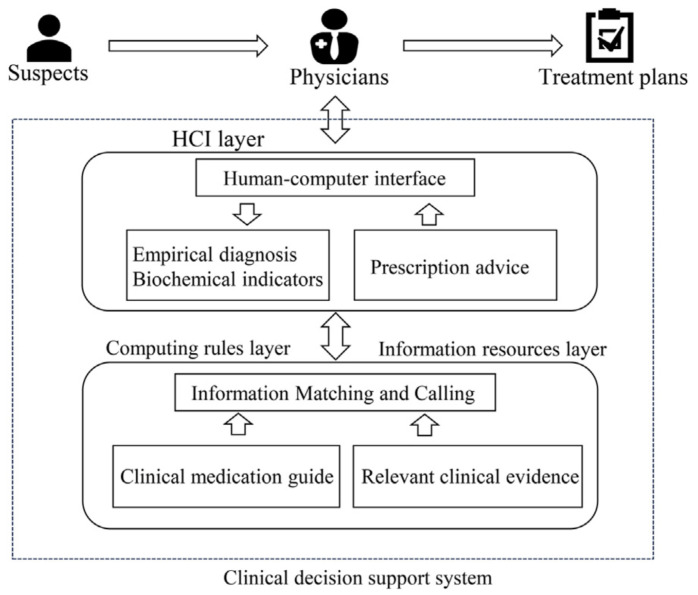
Example of an operational model of a clinical decision support system for antibiotic prescribing [49].

Artificial intelligence-driven decision support systems (AI-DSS) represent a promising approach for optimizing antibiotic use in healthcare settings [48]. These systems leverage advanced algorithms to analyze large amounts of patient data, including medical records, laboratory results, and microbial profiles, thereby supporting healthcare professionals in making prescribing decisions for antibiotics. Figure 4 describes the architecture and operational flow of a clinical decision support system (CDSS) that supports clinicians in antibiotic prescribing decisions by integrating empirical data, guidelines, and clinical trials through a dedicated user interface. The process begins with suspects, i.e., patients with suspected bacterial infections. Information about these patients is then relayed to physicians. The central layer of the model is the human–computer interface (HCI) layer, which serves as the point of interaction between the system and the users (physicians). This level collects empirical diagnoses and biochemical indicators related to patients, and provides advice on antibiotic prescribing. The HCI layer is supported by two underlying layers: (i) the computing rules layer, which performs matching and retrieval of information from clinical drug guidelines and relevant clinical evidence, and (ii) the information resources layer, which actually contains the guidelines and clinical evidence. The output of the system consists of treatment plans for the patients generated based on the collected information and the rules implemented in the CDSS.

A study by Lee et al. [50] demonstrated the feasibility of using machine learning models to predict bacterial or viral infections based on clinical symptoms and laboratory markers, thereby helping clinicians make informed antibiotic prescribing decisions. In addition to hospital-based applications, AI-DSS has the potential to extend antimicrobial stewardship efforts to community-based healthcare providers such as primary care physicians, urgent care clinics, and long-term care facilities [51]. By leveraging telemedicine platforms, mobile applications, and other digital health technologies, AI-DSS can reach a wider audience and empower patients and healthcare providers in decisions regarding antibiotic use [52]. Moffa et al. [53] evaluated the impact of implementing an antimicrobial stewardship program (ASP) in a community teaching hospital on healthcare-associated *Clostridium difficile* infection (HA-CDI) rates and high-risk antibiotic use. Moreover, AI-DSS can support early diagnosis and selection of appropriate antibiotic treatment, improving clinical outcomes for patients. Such a system was described by Lauritsen et al. [54], who presented a deep learning system for early sepsis detection that learns key characteristics and interactions directly from raw event sequence data, eliminating the need for labor-intensive feature extraction. An overview of the potential of AI models in identifying early sepsis to guide antibiotic administration was discussed by Schinkel et al. [55]. However, significant challenges still exist in the effective implementation of AI-DSS for antimicrobial stewardship. One of the most critical issues is the quality and completeness of the data used to train machine learning models, which can be affected by errors, missing values, and biases. Furthermore, many AI algorithms currently used in AI-DSS are black box systems that lack transparency and interpretability, making it difficult for clinicians to understand the underlying rationales behind their recommendations.

## 7. Artificial Intelligence in the Development of Novel Antibacterial Agents

**Figure 5:**
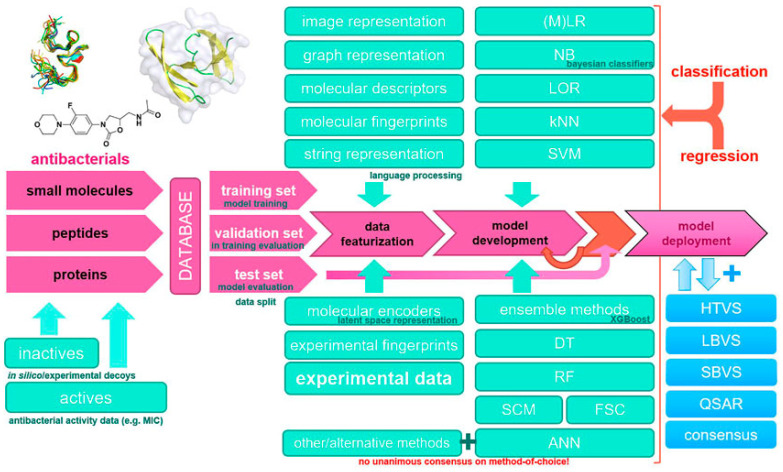
Example of a machine learning methodology and workflow applied to the design of new antibacterial drugs [56].

The emergence of antibiotic-resistant bacteria poses a significant threat to public health, underscoring the urgent need to develop new antibacterial agents. In this regard, AI has emerged as a powerful tool to accelerate drug discovery and development processes, offering innovative approaches for identifying potential antibacterial compounds and optimizing their properties. Figure 5 illustrates a typical machine learning methodology and workflow applied to the design of new antibacterial drugs. The input can consist of different types of compounds, such as small molecules, peptides, proteins, and experimental data on active/inactive compounds and their antibacterial activity, e.g., Minimal Inhibitory Concentration (MICs). These data are divided into training, validation, and test sets and processed through various featurization techniques such as image representations, molecular descriptors, fingerprints, etc. Next, various machine learning methods are applied to develop predictive models, such as Logistic Regression (LR), Naive Bayes (NB), K-Nearest Neighbors (KNN), and ensemble methods. The developed models are then validated and optimized on test data for final use in applications such as virtual screening (e.g., High-Throughput Virtual Screening (HTVS), Ligand-Based Virtual Screening (LBVS), or Structure-Based Virtual Screening (SBVS)), consensus scoring, and new drug design guided by experimental data and Quantitative Structure–Activity Relationship (QSAR) calculations.

Virtual screening, in which machine learning algorithms analyze large databases of chemical compounds to identify molecules with potential antibacterial activity, is a major application area of AI in drug discovery. By training on known antibacterial agents and their structural features, AI models can predict the likelihood of a given compound exhibiting antimicrobial properties, thereby narrowing the pool of candidates for further experimental validation [57]. For example, the use of deep learning algorithms to examine millions of chemical compounds for antibacterial activity has led to the discovery of several new compounds with potent antimicrobial properties [6]. In addition, AI-driven approaches to de novo drug design enable the generation of new molecular structures with optimized pharmacological properties. Generative models, such as Generative Adversarial Networks (GANs) and Variational Autoencoders (VAEs), can generate molecular structures with desired properties such as high potency and low toxicity based on input data from known antibacterial compounds and their structural features [58]. This approach holds promise for the development of new antibacterial agents with increased efficacy and reduced probability of resistance development. AI algorithms can facilitate optimization of lead compounds through structure–activity relationship (SAR) analysis and molecular docking simulations. By analyzing the interactions between potential antibacterial agents and their target bacterial proteins, AI models can predict the binding affinity and efficacy of candidate compounds, guiding the rational design of new antibiotics with enhanced potency and specificity [59]. The research conducted by Ivanenkov et al. [60] used machine learning techniques to optimize the structure of a principal compound targeting bacterial cell wall synthesis, resulting in the development of a potent antibacterial agent with broad-spectrum activity against multidrug-resistant bacteria. In recent years, the role of artificial intelligence has moved beyond the initial identification and optimization of compounds. Machine learning algorithms now help to navigate the complex landscape of pharmacokinetics and pharmacodynamics, predicting a compound’s behavior within biological systems and its efficacy in fighting bacterial infections. By integrating data from preclinical studies and clinical trials, AI-driven models can provide valuable insights into a compound’s safety profile, potential side effects, and optimal dosing regimens, guiding decision-making throughout the drug development process [61,62]. AI holds promise for addressing the challenge of antibiotic resistance through innovative strategies such as optimizing combination therapies and drug repurposing. By analyzing large-scale datasets that include the genetic information of both pathogens and host organisms, AI can identify synergistic drug combinations and reuse existing drugs with known safety profiles for novel antimicrobial applications, thereby maximizing therapeutic efficacy and minimizing the risk of resistance emergence [62].

## 8. Discussion

Antimicrobial resistance represents one of the greatest threats to global public health. This complex phenomenon is due to a combination of biological factors intrinsic to bacteria, such as genetic mutation, as well as external factors related to the misuse of antibiotics in medical, veterinary, and agricultural settings. The rapid and adaptive evolution of pathogens requires a multidisciplinary approach to effectively counter this crisis. The integration of AI in the fight against antimicrobial resistance presents a promising and innovative solution. AI can contribute significantly in several areas:

1. Genomic analysis: AI can accelerate the analysis of genomic data to identify resistance markers early on, thereby improving surveillance and monitoring of resistant infections. This enables timely and targeted interventions. For example, the use of machine learning algorithms to analyze genomic sequences can help to quickly identify specific mutations associated with antibiotic resistance. Such tools can be integrated into clinical microbiology laboratories to provide faster results than traditional methods, allowing clinicians to intervene earlier.
2. Optimizing antibiotic use: AI-based decision support systems can guide clinicians in choosing the most appropriate antibiotics, reducing inappropriate use and minimizing the risk of resistance development. This can significantly improve clinical management of infections. For example, the implementation of a CDSS can help to analyze real-time patient data and microbiological information in order to suggest the most effective therapies while taking into account clinical history and local patterns of resistance. In addition, such systems can be continuously updated with newly collected data to improve their recommendations over time.
3. Discovery of new antibacterial agents: AI facilitates the discovery and design of new antibacterial drugs through predictive modeling and computational simulation, accelerating the drug development process and potentially reducing associated costs. Using deep learning techniques, large libraries of chemical compounds can be analyzed to identify those with potential antibacterial activity. These approaches can also predict the likelihood of success of new drugs at later stages of development, thereby reducing the risks and costs associated with pharmaceutical research and development.
4. AI-controlled delivery and action of antibiotics: AI technologies are increasingly being used to improve the delivery and efficacy of antibiotics. These advanced systems can optimize dosing regimens, improve drug targeting, and monitor patient responses in real time. Significant examples of antibiotics for which administration and action have been successfully managed by artificial intelligence systems include:
  - Optimizing vancomycin dosing with AI: Vancomycin is a key antibiotic for the treatment of serious infections caused by Gram-positive bacteria, including methicillin-resistant Staphylococcus aureus (MRSA). Traditional vancomycin dosing requires careful monitoring to avoid toxicity and ensure therapeutic efficacy. Several studies have shown how AI models can optimize vancomycin dosing to improve efficacy and reduce the risk of toxicity. For example, an approach based on ensemble learning strategies has shown high accuracy and specificity in predicting initial and subsequent doses of vancomycin, making treatment safer and more effective [63,64].
  - AI-driven delivery of amikacin: Amikacin, an aminoglycoside antibiotic, is commonly used to treat severe Gram-negative infections. Its therapeutic window is narrow and requires precise dosing to avoid ototoxicity and nephrotoxicity. Artificial intelligence systems have been developed to monitor blood levels of amikacin in real time and adjust dosing accordingly. These artificial intelligence-driven delivery systems use pharmacokinetic and pharmacodynamic models to ensure that optimal drug concentrations are maintained, thereby improving treatment efficacy and safety. For example, Adbulla et al. [65] conducted a prospective evaluation of a model-based amikacin dosing regimen in infants which showed significant improvements in achieving target drug concentrations compared with traditional methods. Similarly, advances in biosensor technology have enabled real-time monitoring and dose adjustment of antibiotics such as amikacin, leading to improved outcomes in the treatment of critically ill patients [66,67].
  - AI-driven targeted delivery of colistin: Colistin is an antibiotic of last resort for multidrug-resistant Gram-negative bacterial infections; however, its use is limited by significant nephrotoxicity. Researchers have employed artificial intelligence to develop targeted colistin delivery systems, such as nanoparticle-based delivery vehicles, that can be targeted to the site of infection. Artificial intelligence algorithms can optimize the design and release profiles of these nanoparticles to maximize therapeutic effects and minimize systemic toxicity. Early studies indicate that AI-guided targeted delivery significantly reduces adverse effects and improves treatment outcomes. For example, silver nanoparticles conjugated to colistin (Col-AgNPs) have shown enhanced antimicrobial activity and reduced toxicity compared to colistin alone, demonstrating the potential of AI-optimized nanoparticle systems for improving the colistin therapeutic index [68].
  - AI-personalized antibiotic regimens: AI can help to personalize antibiotic regimens by analyzing large amounts of patient data, including genetic information, aiding in the prediction of individual responses to different antibiotics. For example, Zagajewski et al. [69] have demonstrated the use of AI to detect antibiotic resistance and tailor treatments accordingly. This study highlighted rapid detection capabilities and the potential for personalized antibiotic regimens, particularly with ciprofloxacin, showcasing how AI can revolutionize treatment strategies to combat antibiotic resistance. Weaver et al. [70] focused on using reinforcement learning to develop optimal treatment strategies that limit antibiotic resistance. Personalized approaches for various antibiotics, including ciprofloxacin and azithromycin, formed part of their research. A study on personalized dosing of antibiotics at the bedside for severe sepsis and septic shock included ciprofloxacin among the tested antibiotics. Artificial intelligence systems based on pharmacokinetic models were used to optimize dosing, demonstrating potential for improved efficacy and safety in antibiotic administration [71].

Despite significant progress, there are still critical challenges facing the effective implementation of AI in the fight against AMR. One major challenge is the availability of high-quality, properly annotated, and standardized data. In order to develop accurate and reliable machine learning models, it is critical to have large amounts of data representing a wide range of clinical and biological scenarios. However, data from different sources often vary in terms of format, annotation, and quality. This heterogeneity can compromise the performance and generalizability of AI models. Standardizing data and creating centralized repositories with high-quality data are key steps towards improving the effectiveness of AI algorithms in the medical field. Another significant obstacle is ensuring the interpretability and transparency of AI models. Many machine learning algorithms, particularly those based on deep neural networks, are often described as black boxes, as their decisions are difficult to understand and explain. This lack of transparency can create mistrust among healthcare providers and patients, hindering large-scale adoption of these technologies. It is essential that researchers and AI developers adopt explainability practices, such as using interpretable models and implementing post hoc interpretation techniques, to make their algorithms more transparent. Techniques such as LIME (Local Interpretable Model-agnostic Explanations) and SHAP (SHapley Additive exPlanations) [72] can help to explain the predictions of complex models by providing insights into which features most influenced a particular decision. In addition, transparency is critical to ensure that AI models are used ethically and responsibly. Algorithms must be designed to avoid bias and discrimination, which can occur if the training data are not representative of the population or contain bias. Ongoing evaluation and independent validation of AI models are necessary to ensure that they work properly in different populations and clinical settings. Finally, it is important to consider the regulatory and legal aspects of AI use in healthcare. Regulators need to establish clear guidelines for the approval and oversight of AI systems to ensure that they meet standards of safety, efficacy, and privacy. Collaboration among AI developers, healthcare providers, and regulators is essential in order to create an environment of trust and security that facilitates the adoption of AI technologies in the fight against AMR.

Looking forward, the integration of AI with other emerging technologies, such as synthetic biology and nanomedicine, could open up new perspectives in the fight against antimicrobial resistance. For example, AI-based intelligent drug delivery systems could improve the efficacy of antibiotic treatments while reducing the risk of side effects and the development of resistance. In addition, the application of AI to computational epidemiology and predictive modeling could make it possible to anticipate the emergence of new forms of resistance, thereby promoting large-scale prevention and containment strategies. International collaboration is essential to addressing the global challenges of antimicrobial resistance. Sharing data, resources, and knowledge across institutions and countries can improve the effectiveness of AI-based interventions and accelerate progress in combating AMR. In this context, global initiatives such as establishing research consortia and promoting international standards for data collection and analysis can play a key role in overcoming both current and future challenges.
